# The Distribution of Lexical Information Within Dependency Spans: Cross-Linguistic Evidence from 22 Languages

**DOI:** 10.3390/e28101083

**Published:** 2026-09-30

**Authors:** Xiangyu Kang, Shuiyuan Yu

**Affiliations:** School of Linguistics and Language Resources (Institute of Linguistic Sciences), Beijing Language and Culture University, 15 Xueyuan Road, Haidian District, Beijing 100083, China; kangxy2024@163.com

**Keywords:** dependency distance, word surprisal, dependency span, sentence position, lexical composition, cross-linguistic comparison

## Abstract

Dependency distance measures the linear separation between syntactically related words but does not describe how information is distributed across their interval. We analyzed summed XGLM-2.9B word surprisal in 1,346,122 dependency spans from 22 Universal Dependencies languages at distances 4–10. Clustering 154 position-aligned profiles yielded a broadly descending type and a type with a terminal rise; 20 languages retained their assignment across distances. Leave-one-language-out comparison did not support a common three-segment structure: the one-standard-error rule selected two segments for the descending type and three for the rising type. The descending type’s initial decline disappeared after separate adjustments for sentence position and preceding context. Standardizing word-length and model subword-count distributions reduced the rising type’s mean final-word increase from +1.996 to +0.408 bits (95% interval [−0.024, +0.898]). In held-out sentences, real dependencies in eight languages independently classified by a training-set terminal decrease by 0.928 bits more than nearby nonedge intervals sharing the final part-of-speech tag (95% interval [−1.498, −0.217]). A shared part-of-speech component was negatively associated with terminal surprisal in the original descending type. Dependency-span alignment reveals recurring information profiles and locates a conditional terminal contrast for further tests of syntactic prediction.

## 1. Introduction

In dependency grammar, syntactic structure is organized through dependency relations between words. A syntactic dependency is typically represented as an asymmetric binary relation between a head and a dependent, and the difference between their positions in the linear sequence is referred to as dependency distance [1]. Dependency distance provides a concise measure of how far apart two directly related words occur and has been widely used in cross-linguistic comparisons of syntactic structure and in research on linguistic complexity [2,3,4]. Previous research has established a close relationship between dependency distance and syntactic processing demands: longer dependencies generally require comprehenders to maintain more unresolved structural information in working memory. The widespread tendency toward dependency length minimization in natural language has therefore often been interpreted as evidence that linguistic structure adapts to constraints on cognitive resources [5,6,7].

Dependency distance is, however, fundamentally a measure of length. Formally, it reduces the head, the dependent, and the complete linear interval between them to a single distance value, thereby providing a scalar representation of how a dependency unfolds in the linear sequence. Linear intervals containing different lexical items, part-of-speech sequences, and local dependency configurations may have the same dependency distance. Dependency distance thus constitutes a many-to-one mapping: it indicates how far apart two syntactically related items occur but does not preserve the specific sequential composition of the interval between them. Previous studies have recognized the limitations of measuring dependency length solely by the number of intervening words and have incorporated the syntactic properties of the intervening material. In particular, the Intervener Complexity Measure supplements conventional dependency length by quantifying the number of syntactic heads in the intervening region [8,9]. Because dependency distance is frequently used to discuss syntactic processing and cognitive load, it remains important to ask whether length alone adequately characterizes the linear realization of a dependency.

This question can be approached through the contextual predictability of individual words. The probability of a word depends on the preceding context. Psycholinguistic evidence shows that syntactic and semantic information jointly supports the prediction of upcoming words [10], while lexical surprisal, defined in terms of conditional probability, is a robust predictor of reading time [11]. Recent work has also directly linked dependency length to surprisal. Xu and Futrell [12] found that, in most of the languages they examined, the surprisal of the linearly first endpoint of a dependency was positively correlated with dependency length. This result suggests that a less predictable antecedent may receive a more robust memory representation and may consequently support a longer dependency. More recently, Jin and Liu [13] examined the association between dependency distance and surprisal across 34 languages. Their preprint reports that the association varies with dependency direction and relation type: positive associations are more consistent in head-final dependencies, weaker and more variable in head-initial dependencies, and stronger for content dependencies than for functional dependencies. Together, these findings indicate that the linear length of a dependency and the contextual predictability of its component words are not independent, but may reflect a systematic coupling between memory-related and prediction-related demands. They also leave a more fine-grained question open: if the complete interval between a head and its dependent is aligned by relative position, how does surprisal unfold across all the words in that interval, and do stable cross-linguistic distributional patterns emerge?

From an information-theoretic perspective, the surprisal of a word given its preceding context provides a measure of contextual predictability [14,15]. Surprisal has been consistently associated with reading times, eye movements, and neurocognitive measures and is therefore widely used as an index of incremental processing difficulty in language comprehension [16,17,18,19,20]. Previous studies have further shown that information is not distributed uniformly across the linear positions of a sentence: sentence-initial, medial, and final positions may display systematic differences [21,22]. Although the precise information contour of an individual sentence depends on its semantic content and lexical choices, averaging across many observations may therefore reveal relatively stable positional regularities.

Analyses based on whole sentences cannot, however, determine directly whether such regularities are associated with syntactic structure. Most existing studies use absolute sentence position as their reference frame, even though words at the same sentence position may have different syntactic roles and instances of the same dependency relation may occur at different positions. Sentence-based positional analyses consequently have difficulty separating general effects of linear position from syntactically conditioned effects. The present study instead uses the two endpoints of each dependency as structural anchors and aligns the continuous interval between them. This makes it possible to test whether surprisal varies systematically with relative position within a dependency span. The central question is whether the surprisal values across a span merely reflect an accidental aggregation of local lexical properties or form reproducible distributional patterns when many dependencies are considered together.

Answering this question has two main implications. First, it extends the analysis of dependency relations from endpoint distance to the way information unfolds between the endpoints. Second, a cross-linguistic comparison can reveal both the stability of this organization and the sources of its variation. Languages may exhibit recurring contours while differing in their endpoint behavior and in the words and dependency configurations that populate the spans.

We therefore adopt the dependency span as the unit of analysis. A dependency span is defined here as the continuous interval comprising a directly related head and dependent together with every word occurring between them in the linear sequence. Our concern is not whether every individual span follows a fixed trajectory, but whether stable information-distribution patterns emerge after large numbers of comparable spans have been aligned by relative position and statistically averaged.

Using a large pretrained language model, we estimate word-level surprisal and calculate mean surprisal at each position in dependency spans with dependency distances from 4 to 10 in 22 languages. We first examine recurring contour shapes and compare segmented models on the actual observed positions. We then assess sentence position, preceding context, lexical length, and head direction. Finally, we compare the terminal change in real dependencies with nearby same-sentence intervals without a direct dependency between their endpoints and examine one shared axis of part-of-speech composition.

The study addresses three research questions:

1. Does mean surprisal exhibit stable distributional patterns within dependency spans, and are these patterns consistent across dependency distances and languages?
2. How do the observed position profiles and terminal changes vary across languages, and how are they associated with sentence position, lexical composition, and dependency direction?
3. To what extent does the terminal surprisal change in dependency spans differ from that of nearby same-sentence nonedge intervals, and how is this contrast affected by final-word lexical matching and dependency direction?

By addressing these questions, this study evaluates whether the interval defined by a dependency relation provides a useful structural reference frame after accounting for general sentence position and comparison with nearby intervals. It further investigates whether the region traversed by a dependency exhibits statistically organized information patterns and how these patterns relate to syntactic structure, thereby establishing a closer connection between research on dependency distance and information-theoretic approaches to language.

## 2. Materials and Methods

### 2.1. Corpus Data

The syntactic data were obtained from version 2.14 of the Universal Dependencies (UD) treebanks [23]. The set of languages was determined jointly by the availability of UD treebanks and the pretraining coverage of XGLM [24], because all surprisal values were subsequently estimated with XGLM-2.9B. Candidate languages therefore had to occur in the pretraining data of XGLM and in at least one UD v2.14 treebank with the basic HEAD and UPOS annotations needed to construct dependency spans. The analysis used one XGLM-scored language-level CoNLL-U Plus file per language. Corpus sizes in Table 1 count the sentences and integer-ID UD word rows before excluding parts of speech or dependency spans; all qualifying relations within each analyzed file contributed to the corresponding language.

All treebanks were downloaded from the UD website (https://universaldependencies.org) (accessed on 29 September 2026). They provide annotated dependency structures together with word forms, parts of speech, and dependency relations. Table 1 reports the corpus sizes for the 22 languages.

### 2.2. Surprisal Estimation

We use surprisal to quantify the conditional predictability of a word in its preceding context. For the word *x_i_* at position *i* in a sentence, surprisal is defined as the negative logarithm of its conditional probability given the preceding sequence:

(1) $$S(x_{i})-\log_{2}P(x_{i}|x_{1:i-1}).$$

In information-theoretic terms, lower surprisal corresponds to a higher conditional probability and hence to greater model-estimated contextual predictability [14,15,25]. In this study, probabilities are estimated by the multilingual autoregressive language model XGLM from distributions learned during pretraining. The resulting surprisal values primarily represent model-level contextual predictability; they should not be equated directly with human linguistic knowledge, the inherent information value of a word, or real-time human processing load.

Model-derived surprisal is nevertheless relevant to human processing. Previous studies have shown that language-model surprisal explains part of the variation in reading times and eye-movement measures during naturalistic reading, and the relationship between surprisal and reading time has been tested across multiple languages [19,20,26]. These findings provide external-validity evidence for using model surprisal as an estimate of contextual predictability. At the same time, the correspondence is incomplete and model-dependent: lower perplexity or a larger model does not necessarily produce surprisal estimates that better predict human reading behavior, and knowledge acquired from massive training corpora may exceed the experience available to an average human reader [27]. We therefore treat XGLM surprisal as a computational approximation of contextual predictability and restrict our conclusions accordingly. Its relationship to human online processing must ultimately be evaluated with behavioral and neurocognitive data.

Word-level surprisal was estimated with XGLM-2.9B [24], a decoder-only causal Transformer that assigns a conditional probability to the current token on the basis of the preceding token sequence. Its left-to-right objective is therefore directly compatible with the definition in Equation (1). When a word was segmented into multiple subword tokens, the surprisal values of its subwords were summed to obtain word-level surprisal. XGLM was selected both because its autoregressive objective matches the required measure and because it was pretrained under a common architecture on data from 30 languages, allowing the languages in the present study to be analyzed without introducing methodological differences from separate monolingual models. Our sample was drawn from the intersection of the languages covered by XGLM pretraining and those represented by usable UD v2.14 treebanks, thereby limiting additional uncertainty from out-of-coverage inference.

Model diagnostics were computed for UD word tokens with integer IDs in each language before dependency-span filtering; each word was counted once. We report the proportion of words with usable XGLM surprisal estimates, the mean number of model subwords per word, and the proportion of words split into multiple subwords. Subword cross-entropy was calculated by dividing the summed surprisal of scored subwords by their total number, in bits; subword perplexity was calculated as two raised to the power of cross-entropy. Language-specific values are reported in Table A2.

### 2.3. Data Filtering

To reduce the influence of nonlinguistic material on dependency-distance and surprisal estimates, we excluded spans containing any token whose Universal Part-of-Speech tag was PUNCT, SYM, or X. A dependency span was retained only when its head, dependent, and all intervening tokens fell outside these three categories. We excluded the entire span rather than deleting individual tokens and reindexing the remainder, because the latter procedure would shift token positions and break the correspondence between relative positions, part-of-speech distributions, and mean surprisal.

We then retained only dependencies with distances from 4 to 10. Dependency distance was calculated as d = |i − j|, where i and j are the positions of the head and dependent in the sentence. The lower bound was set to 4 because shorter spans contain too few positional observations to characterize the internal distribution at empirical word positions. The upper bound was set to 10 because the number of dependencies decreases rapidly as distance increases, leaving insufficient observations for stable estimates at greater distances. Cross-linguistic research has repeatedly shown that natural languages favor shorter dependencies [2,28,29,30,31,32,33]. In our data, dependencies of length 10 or less accounted for more than 95% of all dependencies in every language. The range from 4 to 10 thus provides enough internal positions for analysis while preserving adequate cross-linguistic sample sizes and stable estimates.

### 2.4. Analysis Overview and Reference Groups

The primary outcome is summed model surprisal per observed word. We use one shared principal component analysis (PCA) [34] to summarize part-of-speech composition and dynamic time warping (DTW) [35] to compare whole-profile shapes. The original raw Type 1 and Type 2 assignments are fixed for descriptive comparisons of the adjusted outcomes. A separate classification by terminal-change sign is trained on one half of the sentences and used to group held-out nonedge comparisons. Unless indicated otherwise, cross-language summaries give each language equal weight, and uncertainty is estimated by resampling complete languages.

### 2.5. Original Profile Classification and Segment Models

The primary all-direction dataset contains one raw word-surprisal curve per language and distance, giving 154 curves. We standardized the d + 1 empirical mean values within each language–distance curve. To avoid counting seven curves from one language as independent replications, the language-level analysis averaged the seven distance-specific DTW dissimilarity matrices before average-linkage clustering. Candidate group counts of two through six were compared with silhouette scores [36], 3000 resamples of 18 complete languages, and 3000 permutations of language correspondence separately within each distance. The labels Type 1 and Type 2 describe the original whole-profile clustering of summed-word surprisal.

Segmented models used the five to eleven actually observed relative-position means on each curve; interpolated display values never entered model fitting. Surprisal was z-standardized within each curve. Within each contour type, the fitted model shared breakpoint locations, with a separate intercept and slope for each segment. We searched breakpoints from 0.10 to 0.90 in steps of 0.05, kept adjacent boundaries at least 0.10 apart, and required every segment to contain at least two distinct empirical positions, ten observations, and data from three languages.

We compared one to five segments at distances 4–10, one to four segments at distances 7–10, and one to five segments at distances 9–10. For K segments, we counted p = 3K − 1 parameters: two regression coefficients per segment and K − 1 breakpoints. The Bayesian information criterion (BIC) [37] was computed as n ln(RSS/n) + p ln(n), with n equal to the total number of observed language-by-distance position means, 517 in original Type 1 and 715 in Type 2. RSS was summed on those same observations.

For leave-one-language-out comparison, we withheld all distance curves from one language and refitted breakpoints and coefficients on the remaining languages. We averaged held-out root mean square error (RMSE) across languages and selected the simplest model within one standard error of the minimum. BIC and predictive choices are reported separately. All terminal analyses use the final two actually observed word positions, irrespective of fitted boundaries.

### 2.6. Sentence Position and Lexical Sensitivity

Relative span position covaries with absolute sentence position and the amount of left context seen by XGLM. We therefore fitted two separate language-specific models. The first predicted summed word surprisal from a piecewise-linear spline of log(1 + absolute word position) and linear and quadratic terms in log(1 + sentence length in words). The second used a spline of log(1 + preceding XGLM subwords), including the beginning-of-sentence token, plus linear and quadratic terms in log(1 + total sentence subword count). Spline knots were at each language’s 10th, 25th, 50th, 75th, and 90th percentiles. Position and context were modeled separately because the two measures are strongly associated.

Each eligible word entered the adjustment fit once, even when it belonged to multiple spans. Five-fold cross-fitting assigned complete sentences, not individual word tokens, to folds; each observation was predicted without using its own sentence. Adjusted word surprisal was observed surprisal minus the out-of-fold prediction plus the language mean. This preserves the bit scale while removing the estimated average sequential trend. Values were mapped back to the same eligible span-position observations and averaged at their original positions.

As a change in information unit, we also divided each word’s summed surprisal by its number of XGLM subwords. Separately, we standardized empirical word positions to the same pooled distribution of subword counts 1–4 and character-length categories 1–3, 4–6, 7–10, and 11 or more within each language, distance, and head direction. Only category combinations with at least five observations at every position supplied common support; words split into more than four subwords did not enter this analysis. Length matching and sequential adjustment are separate analyses with different comparison populations. Fixed original group intervals resampled complete languages 2000 times.

### 2.7. Head Direction and Common Support

We call a span head-initial when its head precedes the dependent and head-final when its head follows the dependent. The 22-language lexical standardization by direction used distances 4–8, where each language had at least 20 spans in each direction and distance. Japanese contributed 27 head-initial spans at distance 8, seven at 9, and one at 10. For direction-specific clustering across all seven distances, we excluded Japanese in both directions, leaving 21 languages with at least 43 spans in each direction–distance cell. We compared raw head-initial, head-final, and equally direction-weighted groupings using DTW. The original all-direction analysis retained all 22 languages.

Terminal comparisons were made separately for each direction while holding the original whole-profile labels fixed. Omitting Japanese from the seven-distance comparison leaves eight consistently Type 1 and eleven consistently Type 2 languages; German and Russian can contribute to 21-language directional reclustering but do not belong to either fixed-label terminal row. Intervals resample whole languages 2000 times. The matched-length directional comparison uses all 22 languages at the distinct supported distances 4–8.

### 2.8. Same-Sentence Nonedge Intervals and Held-Out Analysis

For each eligible real dependency at distance d = 4–10, we sought equal-length windows in the same sentence whose endpoints did not form a direct dependency edge and whose starting word lay within three positions of the real start. Each candidate had to satisfy the same word-score and excluded-UPOS requirements as a real span. For each real span and matching rule, we chose the eligible control with the smallest absolute start displacement. Equally close candidates were resolved by an outcome-independent rule using a fixed seed (20260922) and the sentence and span indices; the accompanying analysis script gives the exact implementation. We repeated selection after additionally requiring the final words to share a Universal Part-of-Speech (UPOS) tag and after requiring both shared final UPOS and the same final XGLM subword count.

A control could be reused by different real spans; no restriction forbade overlap between a matched real and control window. Starts one word apart can cause their last two words to share one token, and their intervening words can substantially overlap. Other direct dependencies may occur within a nonedge window even though its endpoints are not directly related. For each matched pair, we calculated [(Sfinal − Spenultimate)real − (Sfinal − Spenultimate)control], with S the same summed word-surprisal outcome in bits. Final-UPOS and final-subword restrictions select smaller sets of real spans successively, without fixing their exact terminal words or penultimate words.

Complete sentences were assigned to training or test using an eight-byte BLAKE2b digest of the UTF-8 string 20260922|relative-file-path|one-based-sentence-index; a digest interpreted as an unsigned big-endian integer entered training when its value modulo 100 was below 50, and otherwise entered the test set. Thus all candidate spans and windows from one sentence stayed in one half. For each language with at least 100 training nearest-control pairs, the sign of the mean final-minus-penultimate surprisal of its matched real spans defined a decrease or increase group. The real-minus-control contrasts were then estimated on the other half of sentences. For each matching rule, language, and direction, the summary included languages with at least 100 test pairs. Pair totals and mean within-language retained fractions report coverage; 2000 bootstrap draws resampled whole languages for intervals. The held-out sign grouping is distinct from original raw-curve Type 1/Type 2 membership, which was estimated from the full corpus.

### 2.9. Shared Part-of-Speech Composition

For the 20 languages with a consistent original whole-profile classification, we used 1120 observed language-by-distance position means. A single shared PCA used eight mutually exclusive UPOS parts: NOUN, VERB, ADJ, ADV, ADP, DET, AUX, and OTHER. We added a 0.5-count pseudocount to each part, applied centered log ratios, and standardized each component within language before fitting. We oriented PC1 so that noun and verb loadings are positive and checked loading stability by leaving out entire languages. Character length and subword number did not enter the PCA.

For terminal associations, we used the penultimate and final observed positions of each distance curve, fourteen position means per language. We calculated pooled Spearman correlations between shared PC1 scores and raw standardized word surprisal or one of the two separate adjusted outcomes. Partial rank correlations for adjusted outcomes also included mean character length and model subword count. Intervals were resampled from complete languages 1000 times. This position-mean analysis uses neither the lexically standardized curves nor the matched nonedge subset.

## 3. Results

### 3.1. Empirical Profiles and Model Selection

**Figure 1:**
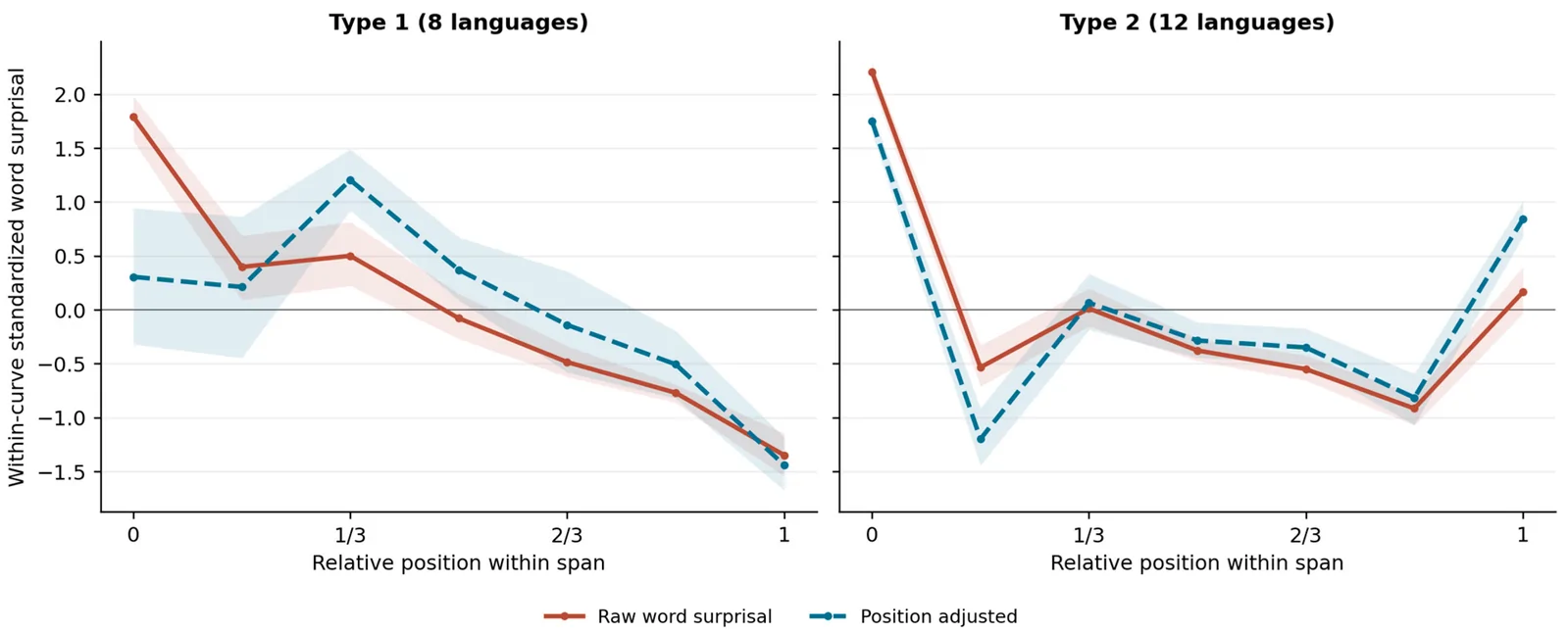
Observed word-surprisal profiles at dependency distance 6 in the eight original Type 1 and twelve original Type 2 languages. The seven observed positions in each d = 6 curve were standardized within their curve before language-equal averaging. Shading gives 95% whole-language bootstrap intervals. The separately position-adjusted series is standardized within each adjusted curve. No interpolation or fitted breakpoints enter this display.

The 1,346,122 eligible dependency spans from 22 languages and distances 4–10 yielded 154 empirical mean word-surprisal curves. The original dynamic-time-warping (DTW) classification placed 65 curves in Type 1 and 89 in Type 2. Type 1 declines broadly across its span and continues downward at the last word; Type 2 descends early, levels over the middle positions, and rises at the last word. Figure 1 shows both raw contours at distance 6 alongside a separately position-adjusted curve. Eight languages retained their Type 1 assignment at all seven distances and twelve retained Type 2; German and Russian changed assignment with distance. The labels describe the shape of the original summed-word outcome, rather than a division of languages by a directly measured grammatical property.

A dependency span includes both directly related endpoints and all intervening words: an endpoint distance d therefore yields d + 1 observed positions. Each curve averages summed-word surprisal at these actual positions, and its ordinate is standardized within the language and distance for comparisons of shape. This construction separates two kinds of observations. Raw bits measure the information assigned to the words present at a position, whereas the standardized contours show how those means vary along the span. The Type 1 and Type 2 profiles are similarly high near their beginnings; their most readily visible difference concerns the final positions. Subsequent bit-scale comparisons therefore use actual adjacent words, without inferring a boundary from visual curvature.

Language-level clustering averaged seven distance-specific DTW dissimilarity matrices so that one language, rather than seven related curves, was the replication unit. Among candidate solutions with two through six groups, the raw two-group silhouette was 0.440 (Table 2). Its assignment had a mean adjusted Rand index of 0.876 across 3000 subsamples of 18 whole languages. Under 3000 permutations that broke the correspondence between a language’s seven distances, a silhouette at least as high occurred with probability 0.00033. Cross-distance language correspondence thus contributes to the observed grouping. The observed advantage of two groups over the best alternative group count was less distinct under the same permutation design (*p* = 0.119), so the descriptive types do not require a sharp boundary between natural language classes.

Of the 65 raw Type 1 curves, 56 come from the eight consistently Type 1 languages; 84 of the 89 Type 2 curves come from the twelve consistently Type 2 languages. The remaining nine Type 1 and five Type 2 assignments come from German and Russian. Table 2 also shows that reclustering the separately position-adjusted and context-length-adjusted outcomes retains a two-group silhouette maximum, with group sizes of 8/14 rather than the raw 9/13. The persistent cross-distance pattern is thus a finding about measured positional profiles, while the exact membership and balance respond to how surprisal is defined and adjusted. Fixed 8/12-language summaries below retain only the original consistently classified languages for clarity.

**Figure 2:**
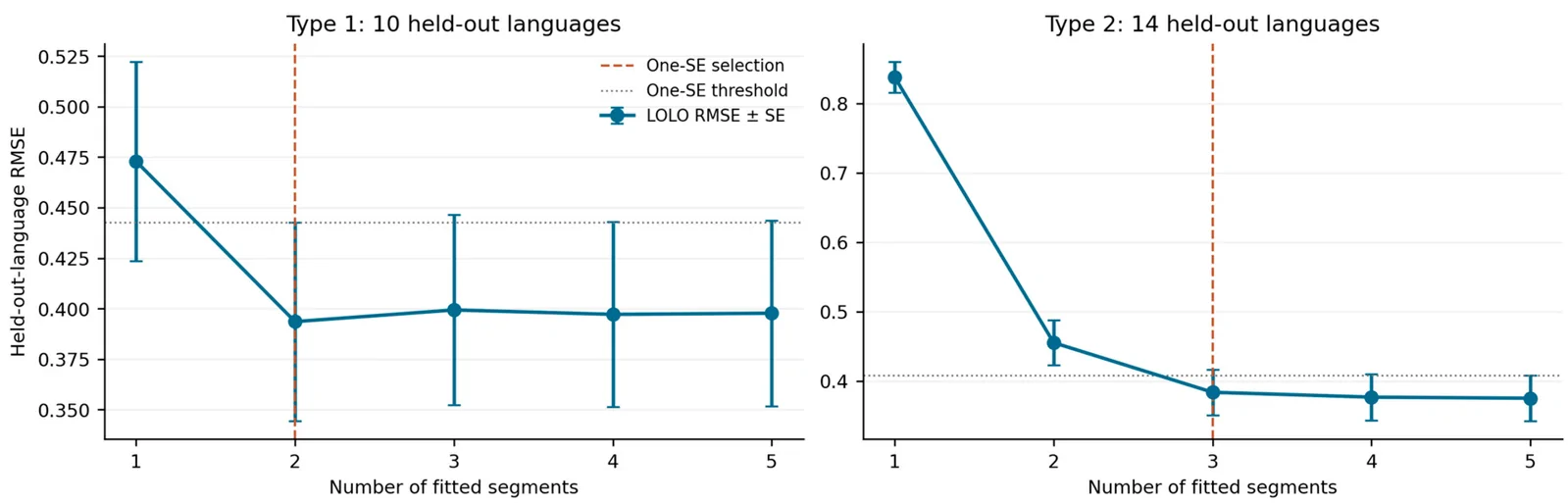
Prediction error for one- to five-segment models of the original empirical position means. Points give mean leave-one-language-out (LOLO) RMSE, with bars showing standard errors across held-out languages. Dashed vertical lines show the most parsimonious model within one standard error of the minimum. Horizontal dotted lines give the one-SE threshold. Type 1 and Type 2 use the original raw-curve assignments, including distance-specific assignments for mixed languages.

The segmented-model comparison used 517 empirical position means from 65 Type 1 curves and 715 means from 89 Type 2 curves; interpolation used to display aligned prototypes entered neither model fitting nor prediction. We searched one through five segments, estimated breakpoints on the training languages in each fold, and predicted all curves of the withheld language. As Figure 2 and Table 3 show, Type 1 reached its minimum held-out-language RMSE at two segments (0.3936), which was also favored by the one-standard-error rule and BIC. Type 2 reached its minimum at five segments (0.3753); its one-standard-error model had three, while its BIC minimum had four. The preferred descriptive complexity differs between the two observed contours.

Adding a third Type 1 segment increased held-out RMSE to 0.3993 and worsened BIC from −935.80 to −930.81. For Type 2, the reduction in RMSE from three to five segments was 0.0086, within the one-standard-error range, whereas BIC preferred four segments to three. The difference between the criteria concerns how finely Type 2 should be divided; none selects three segments as the common preferred model for both types. This matters for the later analyses: shared breakpoints could make different contour shapes appear to have the same number of successive components. We instead compare empirical positions and the final adjacent word pair directly.

Longer distances provide more observed positions with which to check whether the Type 1 two-segment result is driven by five-point curves. Over distances 7–10, the lowest RMSE occurred at four segments for each type; over distances 9–10, the minima occurred at five Type 1 segments and four Type 2 segments. In both restricted ranges, the one-standard-error selections remained two for Type 1 and three for Type 2. The corresponding three-segment RMSEs were 0.4069 and 0.3421 over distances 7–10, and 0.4201 and 0.3274 over distances 9–10. Thus extra subdivisions can improve the point estimate of fit in longer curves, but the parsimonious predictions still do not support a common three-segment structure. The analysis that follows concerns measured sentence position, actual word length, and the terminal contrast.

### 3.2. Sequential Position, Lexical Length, and Direction

**Figure 3:**
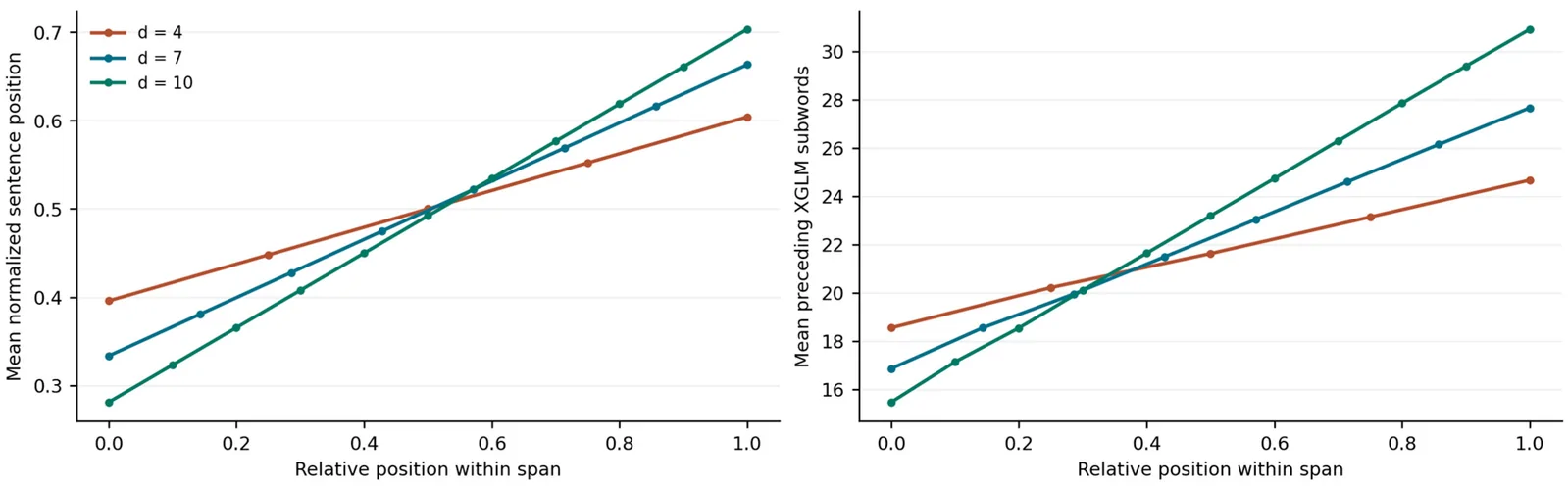
Sentence position and preceding context across actual positions in dependency spans at distances 4, 7, and 10. Each plotted cell averages its 22 language-specific means with equal language weight. The left panel reports the word’s normalized position in its full sentence; the right panel counts model subwords preceding that word. These are covariates, not outcomes adjusted by a segmented model.

The primary outcome remains the sum of model subword surprisals within each observed word, in bits per word. Five follow-up designs ask distinct questions about that outcome. Sentence-position adjustment removes the average trend predicted from a word’s absolute place in its sentence; preceding-subword adjustment removes the average trend predicted from the measured length of the left model context. Dividing by the number of subwords instead changes the information unit, while matching word-length and subword-count categories changes which lexical items contribute to each position. Finally, separating head-initial and head-final spans tests whether different syntactic configurations contribute differently to the raw profiles. Figure 3 displays the sentence-position and preceding-context quantities before adjustment: both increase from earlier to later span positions.

The two sequential adjustments were estimated with five-fold sentence-level cross-fitting on 12,696,032 distinct eligible word tokens; those tokens map into 1,346,122 spans and 8,858,528 span-position occurrences. The language-average out-of-fold R^2^ was 0.3593 in the absolute-position specification and 0.3589 in the preceding-subwords specification. Before adjustment, language-mean correlations of surprisal with their focal predictor were −0.4729 and −0.4849; afterward they were −0.000154 and −0.000109 (Table 4). The two adjusted position-mean outcomes correlated at 0.9989 across 1232 all-direction language-by-distance positions. Their agreement is consistent with the close relation between word position and accumulated context, though each analysis removes only its own measured sequential trend. Every span-position mean was reconstructed from adjusted words, so repeated appearances of a word did not alter the fitting unit.

The original Type 1 profiles lost their most prominent early change under these sequential adjustments. Mean surprisal fell by −2.717 bits from relative positions 0 to 0.25 in the raw Type 1 curves, but changed by +0.379 and +0.374 bits after adjustment for sentence position and preceding subwords, respectively. An early decline appears in the observed lexical sequence, yet its mean is not separate from these general sentence-level trends. At relative positions 0.75–1.00, Type 1 continued to decline: −1.312 bits raw, −1.009 after position adjustment, and −0.927 after context-length adjustment. The corresponding Type 2 changes were +1.523, +1.693, and +1.711 bits. These range-level quantities should be distinguished from the exact last-to-penultimate-word differences in Table 5.

Over the final three observed positions, the fitted raw slope was negative for 55 of 56 curves from the eight consistently Type 1 languages, compared with 51/56 after position adjustment and 49/56 after context-length adjustment. The analogous Type 2 slope was positive in 77 of 84 raw curves and 82/84 under each adjustment. The seven curves of one language share observations and properties, so these counts summarize agreement across dependency distances within language; they are not 56 or 84 independent language tests. The recurrence of opposite terminal directions motivates comparing the real endpoints with an additional same-sentence baseline in Section 3.3.

In the fixed original 8/12-language groups, the final-minus-penultimate mean in the primary summed-word outcome was −0.901 bits for Type 1 and +1.996 bits for Type 2 (Table 5). Standardizing the word observations to shared categories of within-language character length and subword count produced −1.143 bits for Type 1 (95% language-bootstrap interval [−1.426, −0.817]) and +0.408 bits for Type 2 ([−0.024, +0.898]). The alternative per-subword outcome produced −0.807 and +0.396 bits per subword, respectively. This is not a correction to a faulty raw word value: it asks about the mean information of model tokens rather than the total assigned to each word. Similarly, the lexical standardization asks about overlapping categories of words. The original Type 2 rise is an observed property of its actual terminal lexical sample; its estimate among comparable word-length and tokenization categories is smaller and less precise. None of these specifications combines lexical matching with the sequential position adjustments.

Type 2 final words contained, on average, 0.387 more model subwords than the words immediately preceding them. An arithmetic decomposition of its +1.996-bit original word change assigned +1.845 bits to the difference in subword number, +0.581 to mean information per subword, and −0.430 to their covariance. These terms show why total word surprisal, which sums token-level contributions, can rise as the mix of terminal words changes; they do not partition a causal contribution of syntax. Word-length and subword-category standardization address a different, directly comparable population. Moreover, separate reclustering of per-subword profiles produced group sizes of 11/11, whereas reclustering the length-matched profiles gave 21/1. The two raw contour types are useful summaries of the words in the observed spans, with classification details that depend on the information scale.

Head direction also changes the dependency configurations and words in each span. Over distances 4–8, where each language contributes at least 20 spans per distance and direction, head-initial and head-final raw curves clustered into groups of 2/20 and 21/1 languages rather than the balance of the original all-direction types. Japanese illustrates the mismatch between a simple word-order division and the original contour label: 84.6% of its eligible dependencies are head-final, but its raw whole-profile assignment is consistently Type 2. At distances 4–8, the original Type 2 languages had a length-matched terminal change of +0.009 bits in head-initial spans (95% interval [−0.472, +0.535]) and +0.552 in head-final spans ([+0.193, +0.920]). These matched estimates are limited to the 22-language, five-distance comparison in Table 5.

The remaining distances can still be analyzed for other languages: Japanese has 27 head-initial spans at distance 8, seven at 9, and one at 10, whereas every direction-by-distance cell for the other 21 languages at distances 4–10 contains at least 43 spans. In a complete seven-distance directional analysis omitting Japanese in both directions, the raw head-initial, head-final, and equally direction-weighted clustering solutions divided these 21 languages 3/18, 1/20, and 1/20. Fixing the original whole-profile labels, the eight retained Type 1 languages showed final-minus-penultimate changes of −0.749 bits in head-initial spans (95% whole-language interval [−1.011, −0.461]) and −1.006 in head-final spans ([−1.421, −0.525]). For eleven retained Type 2 languages, the corresponding changes were +1.791 ([+1.223, +2.361]) and +2.117 ([+1.686, +2.561]). Table 6 also reports changes after each separate sequential adjustment. Thus, distances 9–10 are retained for 21-language directional comparisons, while the all-direction analysis still includes Japanese and the length-matched directional comparison still uses the supported 22-language distances 4–8. Raw directional clustering is sensitive to which configurations are selected; the fixed-label terminal directions persist in both directions among the included languages.

### 3.3. Real Dependency Spans Versus Nearby Nonedge Intervals

The within-span profiles show where information occurs relative to syntactically related endpoints, but a nearby interval in the same sentence may share a similar late rise or fall. To establish a local baseline, we paired each real dependency span with an equal-length interval in that sentence whose endpoints are not directly connected by a dependency and whose start lies at most three words from the real start. Both spans therefore have the same number of observed word positions and come from similar sentence neighborhoods. For each interval, we measured the summed-word surprisal of the final word minus that of its penultimate word. The real-minus-nonedge difference in these two changes is negative when the real dependency falls more, or rises less, than its paired alternative. This is a comparison of the selected endpoint pair; intervening words in a nonedge interval can still participate in other dependencies.

Formally, the paired statistic is [(Sfinal − Spenultimate)real − (Sfinal − Spenultimate)nonedge], where S is the same bits-per-word outcome in both intervals. We first used the nearest eligible nonedge window. Two additional comparisons required the final words to have the same Universal Part-of-Speech (UPOS) tag or both the same UPOS tag and model subword count. These restrictions address the contribution of broad endpoint lexical composition and tokenization, at the cost of retaining fewer comparable spans. They do not hold the exact final word, penultimate word, or full preceding context identical. The design therefore answers three related questions about different sets of real spans rather than decomposing the raw difference into separate explanatory shares.

In the test sentence halves, nearest-window matching yielded 614,760 pairs across 22 languages and covered a mean of 93.1% of each language’s eligible spans. Giving every language equal weight, the real dependency changed by +1.724 bits more than its paired nonedge interval (95% whole-language bootstrap interval [+0.916, +2.587]; Table 7). Requiring identical final UPOS left 191,141 pairs and 35.3% mean language coverage; the corresponding difference was −0.158 bits ([−0.727, +0.539]). A further requirement of identical final subword count left 94,466 pairs, 18.4% mean coverage, and a difference of −0.082 bits ([−0.571, +0.489]). The large unconditioned difference therefore describes a genuine difference between the observed endpoint samples; in the subsets with a common final POS, the equally weighted overall difference is much less precise and lies near zero.

The change in the mean across matching rules is informative, but its numerical subtraction is not an estimate of the amount caused by part of speech. A UPOS match both holds a category constant and selects a smaller subset of spans, and subword matching reduces that subset again. Whole-language intervals also capture variation among languages; treating hundreds of thousands of paired windows as independent cross-language observations would understate uncertainty. Table 7 therefore displays both the paired count and mean language coverage next to every contrast. These dimensions are essential for evaluating whether an endpoint contrast continues in the population to which the stricter comparison applies.

Languages differed in their direction of terminal change, so we classified that sign on one half of the sentences and estimated every dependency-versus-nonedge contrast on the other half. Eight languages had a final-word decrease in the training half, and fourteen had an increase; all 22 retained their training sign in the test half. This decrease/increase division is separate from the original Type 1/Type 2 labels fitted to complete raw word-surprisal curves. Chinese belongs to the original Type 1 but has an increasing held-out endpoint sign; German has mixed original whole-profile assignments yet enters the training-decrease group, and Russian enters the increase group. Holding the classification rule apart from the test sentences gives the endpoint comparison a clearer interpretation than reusing its own test signs to define the groups.

**Figure 4:**
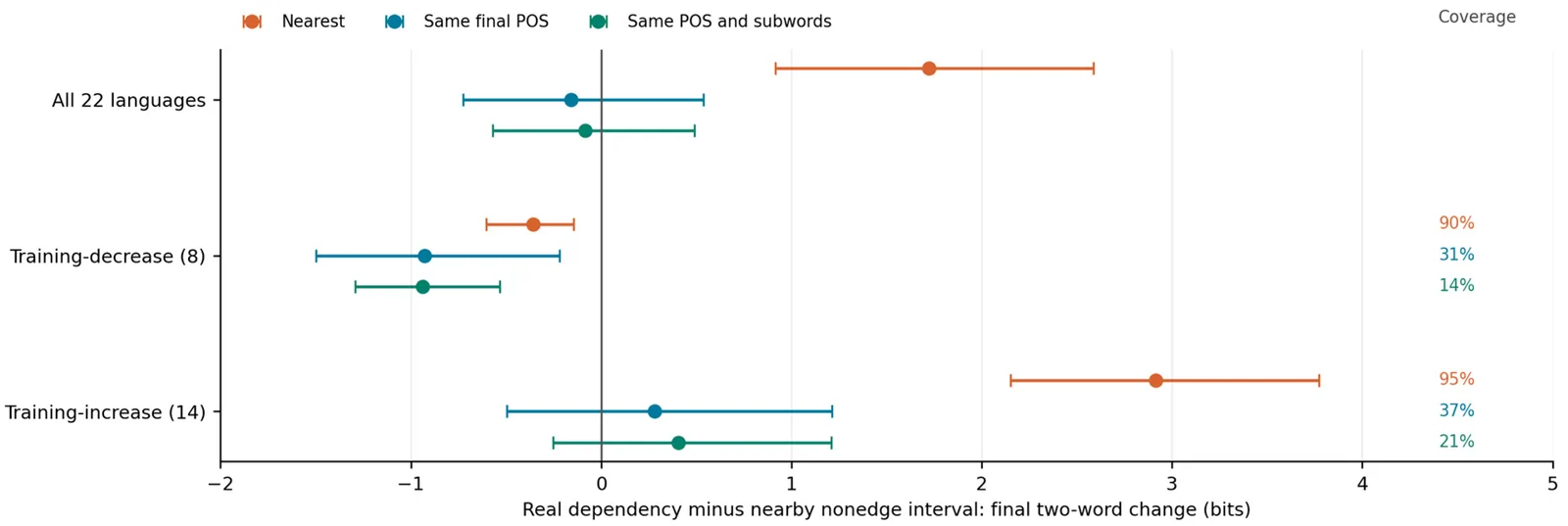
Held-out final-two-word contrast between real dependency spans and nearby same-sentence, equal-length nonedge intervals. Points are means that give each language equal weight; bars are 95% bootstrap intervals resampling entire languages. Training sentence halves determine the decrease/increase labels; plotted estimates use test sentence halves. The right-side percentages show mean language coverage in the two sign groups. The three matching rules select different populations.

In nearest-window matches, the real-minus-nonedge test estimate was −0.357 bits for the eight training-decrease languages (95% interval [−0.604, −0.145]) and +2.913 for the fourteen training-increase languages ([+2.153, +3.773]; Figure 4). Matching final UPOS left −0.928 bits ([−1.498, −0.217]) in the decrease group and +0.282 ([−0.494, +1.212]) in the increase group. With final subword count matched as well, the estimates were −0.937 ([−1.292, −0.533]) and +0.406 ([−0.254, +1.209]) bits, respectively. Under the final-UPOS rule, the between-group difference in real-minus-nonedge estimates was −1.210 bits (interval [−2.229, −0.175]). Coverage averaged 31.5% of eligible spans in the decrease group and 37.4% in the increase group; the stricter match retained 14.0% and 20.9%. The test thus finds an additional terminal decline in a restricted comparable-category subset of decreasing languages, while the positive conditional estimate in the increasing languages remains uncertain.

The asymmetry matters for the interpretation of both original contours. An increase in the total surprisal of actual terminal words can coexist with little measurable excess over nonedge intervals among pairs ending in the same broad category. Conversely, a real dependency ending in a decrease can decline even more than its paired nonedge window among available same-UPOS words. The latter contrast concerns a conditional set of spans, not all dependency edges in a language. Pairing within sentences restricts some global differences in text and sample, yet the two words at the end may still differ in lemma, morphology, syntactic role, and detailed preceding context. The estimate is accordingly evidence for where a closer structural test should focus: the terminal word pair among syntactically anchored intervals.

A separate sensitivity summary groups the same held-out pairs by their original whole-profile labels (Table 8). In the eight consistently Type 1 languages, the nearest-window estimate is −0.101 bits with an interval containing zero, whereas the final-UPOS-matched and UPOS-plus-subword estimates are approximately −0.791 and −0.926 bits. In the twelve consistently Type 2 languages, the nearest-window estimate is +3.269 bits and the corresponding restricted estimates are about +0.341 and +0.443 bits, each with an interval containing zero. This similarity to the endpoint-sign comparison locates the link between the original curve shapes and the more direct local test. The two classifications do not have identical membership, and the original whole-profile labels were estimated on the full raw-curve sample. The held-out endpoint grouping in Figure 4 and Table 7 provides the independently assigned sign comparison.

### 3.4. Shared Part-of-Speech Composition at the Actual Endpoint

**Figure 5:**
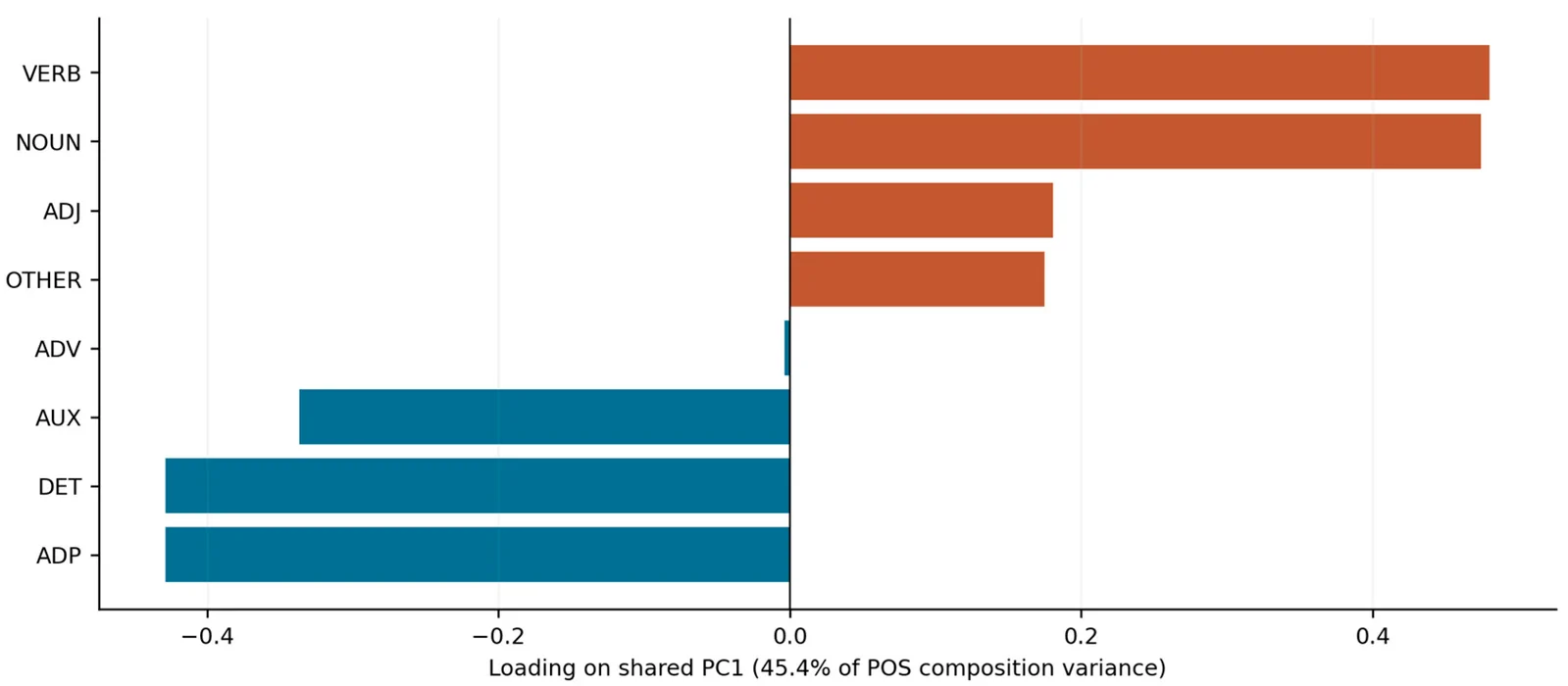
Loading direction of the single shared UPOS-composition PC1 fitted to 1120 empirical position means. The eight mutually exclusive components are NOUN, VERB, ADJ, ADV, ADP, DET, AUX, and OTHER; PC1 is oriented for positive noun and verb loadings. Loadings describe relative category composition rather than the surprisal of any individual tagged word.

The 20 languages consistently assigned to one original whole-profile type supplied 1120 actual language-by-distance position means for a single shared PCA of eight mutually exclusive UPOS parts. After centered log-ratio transformation and within-language standardization, PC1 explained 45.4% of their compositional variance. VERB (+0.481) and NOUN (+0.475) had their largest positive loadings; ADP (−0.429), DET (−0.428), and AUX (−0.337) loaded negatively (Figure 5). Re-estimating the PCA while omitting one language at a time gave loading-vector cosines of 0.99918–0.99995. The resulting scores describe how the relative mixture of word categories changes over empirical positions in one common coordinate system, even when the two original raw contour types differ.

Each mean position contributes the shares of NOUN, VERB, ADJ, ADV, ADP, DET, AUX, and OTHER, with a 0.5-count pseudocount before taking log ratios. Character length, subword number, and derived content-word fractions do not enter the PCA; the first two are instead used when examining their association with surprisal. PC1 is oriented so that NOUN and VERB have positive loadings, making the sign of its correlation readable; reversing this arbitrary axis would reverse all reported signs without changing the relationship. The remaining components describe other directions of POS-composition variation, but the following comparison concerns only this same PC1 at each actually observed penultimate and final position. It does not assign components to fitted segments.

At the last two empirical positions of every curve, each language contributes fourteen distance-position means. Table 9 reports pooled Spearman PC1–surprisal associations separately in the eight original Type 1 and twelve original Type 2 languages, with language-bootstrap intervals. The adjusted comparisons use either position-adjusted or context-length-adjusted word surprisal, followed in each case by rank-based partialing of mean character length and model subword count. This is a position-mean analysis of the observed lexical samples. It neither reweights those words to the common lexical-length categories in Table 5 nor reproduces the restricted real-versus-nonedge pairs of Table 7. Its role is to test whether a common POS-composition axis has the same empirical relationship to the original type-specific terminal profiles.

In the original Type 1 group, the pooled last-two-position raw association was approximately −0.473; it became −0.727 with separate position adjustment and length partialing, and −0.741 with the corresponding preceding-context adjustment. The original Type 2 association was strongly positive in raw means (+0.807) but near zero after either adjusted-and-length-partial specification (−0.043 and −0.044, with whole-language intervals spanning zero). Seven of the eight Type 1 languages had negative within-language raw and position-adjusted length-partial terminal associations; Chinese was the exception. These results connect the observed descending profile to which broad word categories occur near its endpoint, while showing that a stable PCA loading direction need not imply the same surprisal association for the other original contour type. Distances and positions remain repeated observations within each language.

A descriptive within-POS comparison also found lower raw surprisal for many verb heads than for dependent or span-internal verbs in the original declining profiles. Because the particular verbs, their model tokenization, and preceding contexts were not matched across roles, this observation does not establish the source of the paired endpoint contrast in Section 3.3.

## 4. Discussion

### 4.1. Positional Information Within Dependency Spans

Dependency distance records the separation of a head and dependent, whereas the aligned span records the surprisal assigned to every word between them. The recurring raw curves show that dependencies of equal distance can be compared in terms of a positional information profile: the original Type 1 curve descends broadly and continues downward at the end, while Type 2 falls, levels, and ends in a rise. The language-level grouping recurs across distance, so these shapes are more than isolated properties of one span length. Yet the empirical segment comparison gives them different parsimonious complexities, and the initial Type 1 descent disappears after either measured sequential adjustment. The most informative recurring difference is therefore near the observed right endpoint. Treating these positions as observations rather than successive universal phases keeps the descriptive regularity and identifies where an additional structural comparison is possible.

The raw profiles and the local nonedge test answer related but distinct questions. A mean dependency-span curve describes how the actual sampled words distribute model surprisal as one moves from the left endpoint to the right. The local test asks whether the last adjacent pair changes differently from a nearby same-sentence word interval of the same length whose endpoints are not a direct dependency edge. In this dataset, the original whole-profile clustering is reasonably stable across seven distances, but that fact alone does not demonstrate a property unique to dependent–head endpoint pairs. The nonedge baseline puts the contrast on a comparable local sequence and reveals how much the answer changes when the final word belongs to the same broad grammatical category. Dependency-span alignment thus provides a useful structural frame for locating information; the paired comparison assesses the specificity of its endpoint pattern.

The first part of the curves must also be read in light of the sentence from which each span was drawn. Later span positions occupy later absolute sentence positions and follow more XGLM subwords. Removing the average influence of either measured sequence variable makes the early Type 1 fall disappear, whereas the late decline or rise remains visible in the original fixed groups. This difference between early and late behavior helps explain why an aligned curve can look internally ordered without requiring a common segmentation mechanism. In particular, assigning three fitted stages would conceal the fact that the evidence for an early stage and for a terminal difference comes from different comparisons. The terminal observations can instead be examined in their own word-level units and against explicit nearby alternatives.

### 4.2. Asymmetric Sensitivity of Descending and Rising Endpoints

The asymmetry between terminal directions is the principal empirical finding. In original Type 2 spans, the words actually present at the right endpoint carry more summed model surprisal than their immediate predecessors. They also contain more subwords on average, and the rise is much smaller when length and tokenization categories contribute in similar proportions at both positions. In the held-out increase group, the large unconditioned real-minus-nonedge rise becomes imprecise when both final words share a UPOS tag. These observations place word-category composition and head-direction-related sampling inside the explanation for the observed Type 2 contour. A naturally occurring increase in the information of endpoint words is still a real distributional property; it simply answers a broader question than whether a terminal contrast persists when the lexical properties of both terminal positions are more closely equated.

The descending pattern remains more specific under the checks available here. The original Type 1 last-two-word decrease continues after separate adjustments for sentence position or measured preceding context and in the distribution-standardized word sample. More directly, languages placed in the decrease group on training sentences have about 0.9 bit more terminal decline in real spans than in nearby nonedge intervals among test pairs with a common final UPOS. Requiring the same final model subword count retains a similar negative estimate, although fewer spans remain. This is a concrete departure from the local baseline in the portion of the sample that supports matching; it offers a more precise target for explaining why the final two positions of certain dependency configurations exhibit a stronger decline. The result does not require every declining whole curve to be a member of the held-out decrease group, since the two groupings use different definitions.

The shared POS component helps locate composition within this asymmetry. Its positive noun and verb and negative adposition, determiner, and auxiliary loadings define one stable coordinate for all observed positions, and its negative terminal association in original Type 1 means survives the examined sequential adjustments with partial control for mean word and subword length. The original Type 2 positive raw association largely vanishes under the same comparisons. PCA is an aggregate description of word categories at position means; matching final UPOS between two windows conditions individual endpoints. The negative Type 1 aggregate association and the negative within-UPOS local contrast can therefore coexist. Their joint pattern favors testing terminal composition and individual lexical identity together, rather than treating one analysis as an automatic explanation of the other.

### 4.3. Sentence-Level Information, Dependency Endpoints and Prediction

Research on the distribution of information over sentence positions shows that the amount or predictability of material can change as a sentence unfolds [21,22]. Those findings and the present analysis address different alignments: sentence studies start from the whole sentence, while our curves start from the two endpoints of a syntactic relation. Their connection is directly visible in the covariates. Span-relative position moves with absolute sentence position and accumulated model context, and the raw early Type 1 drop no longer has the same direction after these general trends are removed. Part of the span curve therefore reflects ordinary sequential organization. Identifying that shared component makes the remaining terminal pattern a sharper empirical problem rather than treating every dip and rise inside a span as a direct consequence of syntax.

The endpoint literature supplies a complementary reference point. Xu and Futrell [12] related the surprisal of a dependency’s linearly first endpoint to dependency length; Jin and Liu [13] report direction- and relation-dependent associations between distance and surprisal in their cross-language preprint. These studies establish that the information at dependency endpoints varies with properties of the relation, but they do not describe every intervening position. Our aligned curves fill that descriptive gap, and the local comparison brings attention back to the final two positions as the clearest locus for an additional contrast. The right endpoint of a span need not be the linearly first endpoint in earlier studies, and it may be either head or dependent depending on direction. This distinction prevents an immediate jump from the observed late decline to a single grammatical-role explanation.

A second approach to comparison places groups in a common latent representation. For example, Khalid et al. [38] use pooled semantic dictionary learning to quantify overlap among mental-health text conditions. Here, DTW compares the shapes of positional surprisal curves, while the single shared POS-composition component places languages on one interpretable axis of relative word-category frequencies. That component is neither a semantic dictionary nor an independent measure of typological similarity. Future work could model latent lexical and syntactic composition jointly with information trajectories to test which between-language differences remain after common features are represented explicitly.

Expectation-based accounts propose that preceding material can constrain the identity or structural category of an upcoming word, and work on locality and antilocality examines when added preceding material helps or hinders comprehension [14,15,39,40,41,42]. If a real dependency span exhibits a stronger final-two-position decline than a matched local nonedge interval, accumulated predictive constraints are one possible account of that contrast. The comparison now gives that interpretation a concrete target: the final two words of spans in held-out decreasing languages, within the pairs that share a final UPOS and sometimes subword count. It does not measure the information retained about the dependency itself or keep the exact lexical target and preceding history constant. This is why the evidence motivates a prediction-based test without uniquely identifying predictive facilitation by the dependency relation.

A closer test would hold the same final target word, or at least its lemma and relevant morphological features, across alternative preceding contexts. It would also align or explicitly account for the penultimate word, the location in the sentence, dependency relation type, and the information in the accumulated left context. Such a design would distinguish more effectively between a predictable endpoint because of the relation being completed and a predictable endpoint because particular words, constructions, and sentence neighborhoods populate the selected span type. The present difference supplies the sign, location, and subgroup for that test. Retaining the observed Type 2 lexical rise alongside the narrower Type 1 conditional decline prevents a single expectation mechanism from being imposed on two empirically different patterns.

### 4.4. Limits of the Present Comparison

All word probabilities were estimated with XGLM-2.9B on the selected UD treebanks. Different language tokenization affects the number of subwords summed into word surprisal, and character count is not a morphologically equivalent unit across writing systems. The separately cross-fitted position and preceding-context adjustments, lexical-length standardization, and local nonedge matching examine distinct sources of variation; no single estimate simultaneously equates all of these properties. Type 1 and Type 2 labels derive from the original full-sample curves, while the decrease/increase sign labels for the local analysis were assigned on training sentences and evaluated on test sentences. These distinctions limit comparisons across columns but are explicit in the results and tables.

The same-UPOS real-minus-nonedge result for the decrease group covers 31.5% of eligible spans on average, falling to 14.0% when final subword count is also matched. A nearby nonedge interval can contain other dependencies, and the paired windows may overlap with a real span, even sharing one terminal-pair token when their starts differ by one word. They need not share the same final target, penultimate word, or entire preceding context. The approximately −0.9-bit difference combines differences at the final and penultimate positions; the available aggregate files cannot attribute it to either position or recalculate it after excluding shared terminal-pair tokens. These limitations place the observed approximately −0.9-bit difference in a restricted supported sample rather than in all dependency spans. Repeating a more tightly matched analysis in another treebank and with another multilingual model would test the stability of the pattern; behavioral data would be needed before interpreting model-estimated surprisal as a directly observed human processing advantage.

## 5. Conclusions

Dependency distance reduces the linear separation between a head and its dependent to a single scalar value. This study instead examined the information assigned to all words across the interval defined by these two endpoints in 1,346,122 spans across 22 languages, and compared the terminal change in real dependencies with nearby equal-length nonedge intervals from the same sentence. The span alignment reveals recurring positional organization that a distance statistic alone does not display. The paired baseline locates which part of that organization remains distinctive when alternative endpoint pairs and final-word categories are considered.

In the original summed-word outcome, Type 1 generally declines and continues to fall at the end; Type 2 falls early, levels, and rises at the end. Empirical model comparisons do not support a common preferred three-segment structure. The Type 1 early decline also disappears after separate adjustments for general sentence position and measured preceding context. Late directions persist in the original fixed groups, but Type 2’s approximately +2-bit raw last-word increase becomes about +0.4 bit with an interval crossing zero when word-length and model subword-count distributions are standardized. Its observed rise therefore reflects, in substantial part, the words that occur near the endpoint.

The local nonedge comparison shows a further asymmetry. The unconditioned 22-language real-minus-nonedge terminal difference is +1.724 bits; among pairs sharing final UPOS, its mean is near zero with a broad interval. In training-defined decreasing languages, however, real spans in held-out sentences show about 0.9 bits more terminal decline than nearby nonedge intervals among the restricted same-UPOS endpoint pairs, with a similar difference after matching final subword count. The training-defined increasing languages do not show an equally precise positive residual under those comparisons. One shared POS component also has a negative association with original Type 1 terminal surprisal under the examined adjustments, while Type 2’s strong raw positive association attenuates. These findings identify where lexical composition and the structural choice of endpoint pair each matter.

The conditional terminal decline is the paper’s strongest candidate for an additional dependency-related information pattern: a specific location, direction, and matched subgroup can now be tested with fixed lexical targets and more closely equated contexts. Its compatibility with an expectation-based account is a productive hypothesis rather than an established causal mechanism. Comparing independent corpora, other multilingual models, and human comprehension measures will show whether the difference survives stronger lexical and contextual matching and whether it generalizes beyond the present supported sample.

## Figures and Tables

**Table 1:** Sentences and integer-ID UD words in the analyzed language-level files.

Language Family	Language	Tokens	Sentences
Indo-European (Germanic)	German	3,810,131	208,438
	English	742,304	46,310
Indo-European (Romance)	Catalan	547,261	16,678
	Portuguese	1,464,445	78,141
	Spanish	1,023,080	35,214
	French	635,081	29,735
	Italian	879,629	37,871
Indo-European (Slavic)	Russian	1,830,033	111,238
	Bulgarian	156,149	11,138
Indo-European (Indo-Aryan)	Urdu	138,077	5130
	Hindi	351,704	16,649
Indo-European (Hellenic)	Greek	88,934	4328
Uralic (Finnic)	Finnish	396,999	36,981
	Estonian	528,411	38,147
Turkic	Turkish	681,936	64,673
Sino-Tibetan (Sinitic)	Chinese	186,619	9947
Austronesian	Indonesian	169,728	7628
Austroasiatic	Vietnamese	59,957	3423
Afro-Asiatic (Semitic)	Arabic	212,751	5608
Language isolate	Basque	121,443	8993
Japonic	Japanese	222,442	9100
Koreanic	Korean	446,996	34,702

**Table 2:** Clustering diagnostics for the original word outcome and two separately adjusted outcomes.

Outcome	Language k = 2 Silhouette	Group Sizes	18-of-22 Subsample ARI
Raw summed word	0.440	9/13	0.876
Position adjusted	0.491	8/14	0.987
Context adjusted	0.473	8/14	0.996

Note. Whole-language clustering averages distance-specific DTW matrices before comparing k = 2–6. At the 154-curve level, the respective silhouette values for k = 2, 3, 4, 5, and 6 on the raw outcome are 0.4036, 0.3589, 0.3240, 0.3283, and 0.3352. ARI is the mean adjusted Rand index of 3000 18-language subsamples. The adjusted outcomes were modeled separately, and the group sizes are not the original eight/twelve stable-language groups.

**Table 3:** Empirical-position model comparison across distances 4–10.

Original Curve Type	Segments	LOLO RMSE (SE)	BIC
Type 1	1	0.4729 (0.0494)	−743.69
	2	0.3936 (0.0491)	−935.80
	3	0.3993 (0.0471)	−930.81
	4	0.3972 (0.0458)	−919.22
	5	0.3978 (0.0460)	−901.53
Type 2	1	0.8376 (0.0224)	−228.42
	2	0.4553 (0.0326)	−1050.35
	3	0.3839 (0.0330)	−1368.02
	4	0.3769 (0.0336)	−1384.57
	5	0.3753 (0.0326)	−1375.18

Note. Lower RMSE and lower BIC indicate a better fit under different criteria. The Type 1 and Type 2 analyses comprise 517 and 715 actual position means from 65 and 89 curves, respectively. They use 10 and 14 distinct held-out languages because mixed German and Russian contribute to both assignments; no interpolated DTW prototype points enter model selection.

**Table 4:** Sentence-position and preceding-context adjustment diagnostics across 22 languages.

Separate Adjustment	Mean Out-of-Fold R^2^	Mean Raw r	Adjusted r/Max. |r|
Absolute word position	0.3593	−0.4729	−0.000154/0.001409
Preceding model subwords	0.3589	−0.4849	−0.000109/0.001478

Note. The focal association compares model-estimated word surprisal with the stated predictor in each language. The table reports unweighted means across language-specific token models; the last number is the largest absolute adjusted correlation in a single language. Nuisance adjustment was carried out on eligible words before the word outcomes were re-aggregated by span position.

**Table 5:** Last-two-position change in original and lexical-length-matched word surprisal.

Direction/Distances	Original Group	Word Bits	Matched Word Bits [95% CI]
All/4–10	Type 1	−0.901	−1.143 [−1.426, −0.817]
All/4–10	Type 2	+1.996	+0.408 [−0.024, +0.898]
Head-initial/4–8	Type 1	−0.793	−1.118 [−1.359, −0.825]
Head-initial/4–8	Type 2	+1.454	+0.009 [−0.472, +0.535]
Head-final/4–8	Type 1	−0.969	−1.180 [−1.486, −0.839]
Head-final/4–8	Type 2	+2.109	+0.552 [+0.193, +0.920]

Note. Each entry is a final-minus-penultimate mean. The last column standardizes to shared character-length and 1–4-subword categories with sufficient observations at each relative position; whole languages were resampled 2000 times. The unmatched per-subword outcomes are −0.807 and +0.396 bits/subword for the all-direction Type 1 and Type 2 groups, respectively, and have different units from the table’s word-bit columns. The fixed original grouping is conditional on the raw outcome.

**Table 6:** Terminal changes by head direction over distances 4–10 in the 19 consistently classified non-Japanese languages.

Direction/Original Type	Raw Word Bits [95% CI]	Position Adjusted [95% CI]	Context Adjusted [95% CI]
Head-initial/Type 1 (8)	−0.749 [−1.011, −0.461]	−0.587 [−0.860, −0.290]	−0.545 [−0.833, −0.251]
Head-initial/Type 2 (11)	+1.791 [+1.223, +2.361]	+1.875 [+1.308, +2.431]	+1.878 [+1.341, +2.474]
Head-final/Type 1 (8)	−1.006 [−1.421, −0.525]	−0.824 [−1.260, −0.332]	−0.770 [−1.179, −0.255]
Head-final/Type 2 (11)	+2.117 [+1.686, +2.561]	+2.237 [+1.823, +2.640]	+2.250 [+1.846, +2.661]

Note. Values are final-minus-penultimate word surprisal in bits. Original whole-profile type labels are fixed; German and Russian are excluded from type rows. Every distance from 4 to 10 is included. Intervals resample whole languages 2000 times. Adjustments are separate and do not match lexical length.

**Table 7:** Same-sentence nonedge control: test-set language-equal terminal contrasts.

Match/Training Sign Group	Real Minus Control [95% CI], Bits	Pairs	Mean Coverage
Nearest/All 22	+1.724 [+0.916, +2.587]	614,760	93.1%
Nearest/Dec. 8	−0.357 [−0.604, −0.145]	332,213	90.5%
Nearest/Inc. 14	+2.913 [+2.153, +3.773]	282,547	94.6%
Final POS/All 22	−0.158 [−0.727, +0.539]	191,141	35.3%
Final POS/Dec. 8	−0.928 [−1.498, −0.217]	88,101	31.5%
Final POS/Inc. 14	+0.282 [−0.494, +1.212]	103,040	37.4%
POS + SW/All 22	−0.082 [−0.571, +0.489]	94,466	18.4%
POS + SW/Dec. 8	−0.937 [−1.292, −0.533]	36,707	14.0%
POS + SW/Inc. 14	+0.406 [−0.254, +1.209]	57,759	20.9%

Note. Dec./Inc. are training-set endpoint signs; all estimates use test sentences and resample languages. POS + SW matches final UPOS and model subword count. Coverage is the mean language-specific retained fraction; control windows may be reused.

**Table 8:** Sensitivity of the held-out local comparison to the original whole-profile labels.

Match/Original Group	Real Minus Control [95% CI], Bits	Pairs	Coverage
Nearest/Type 1	−0.101 [−0.352, +0.236]	120,518	90.4%
Nearest/Type 2	+3.269 [+2.617, +4.072]	232,398	95.8%
Final UPOS/Type 1	−0.791 [−1.421, −0.121]	45,807	34.6%
Final UPOS/Type 2	+0.341 [−0.563, +1.395]	84,790	37.6%
UPOS + subwords/Type 1	−0.926 [−1.287, −0.523]	21,201	16.5%
UPOS + subwords/Type 2	+0.443 [−0.363, +1.381]	48,532	21.0%

Note. Only the 20 languages with consistent original DTW labels are included. The original labels were obtained from the full raw-curve dataset, including the sentences represented here, and are descriptive conditioning rather than an independently held-out classification. Intervals are from 2000 whole-language bootstrap resamples; group summaries weight languages equally. German and Russian are excluded.

**Table 9:** Shared PC1 associations with word surprisal in the last two observed positions of each curve.

Original Group	Raw Rho [95% CI]	Position + Length Partial Rho [95% CI]	Context + Length Partial Rho [95% CI]
Type 1 (8 languages)	−0.473 [−0.706, −0.259]	−0.727 [−0.872, −0.405]	−0.741 [−0.877, −0.450]
Type 2 (12 languages)	+0.807 [+0.744, +0.866]	−0.043 [−0.215, +0.220]	−0.044 [−0.222, +0.217]

Note. The PCA loadings were fixed from all 1120 empirical position means; the displayed correlations use 112 Type 1 and 168 Type 2 final-two-position means. Each language contributes 14 correlated position means over distances 4–10. Sequential adjustments were fitted separately; both partial columns also control mean word length and mean subword count. Intervals resample whole languages 1000 times. The original grouping was selected from these source curves.

## Data Availability

The UD treebanks are available from the Universal Dependencies project. The project repository is https://osf.io/qf8h9/overview (accessed on 29 September 2026). Scripts and aggregate result tables for the additional analyses accompany the revised manuscript as a supplementary analysis archive. The language-level XGLM-scored input files are available from the corresponding author upon reasonable request.

## Appendix A. Original Cross-Distance Curve Assignments

**Table A1:** Original word-surprisal type assignments across distances 4–10.

Language	Type 1 (of 7)	Type 2 (of 7)
Urdu	7/7	0/7
Russian	3/7	4/7
Bulgarian	0/7	7/7
Catalan	0/7	7/7
Hindi	7/7	0/7
Indonesian	0/7	7/7
Turkish	7/7	0/7
Basque	7/7	0/7
Greek	0/7	7/7
German	6/7	1/7
Italian	0/7	7/7
Japanese	0/7	7/7
Chinese	7/7	0/7
French	0/7	7/7
Estonian	7/7	0/7
Finnish	7/7	0/7
English	0/7	7/7
Portuguese	0/7	7/7
Spanish	0/7	7/7
Vietnamese	0/7	7/7
Arabic	0/7	7/7
Korean	7/7	0/7

Note. Each language contributed seven curves, one at each dependency distance from 4 to 10. The numbers indicate the curves assigned to each original type.

## Appendix B. Model Tokenization Diagnostics

**Table A2:** XGLM word coverage and subword tokenization across 22 languages.

Language	UD Words	Usable Estimates (%)	Subwords per Word	Multi-Subword Words (%)	Cross-Entropy (Bits/Subword)	Perplexity
Arabic	212,751	100	1.46	36.6	5.36	41.2
Basque	121,443	100	1.93	67.3	6.52	91.7
Bulgarian	156,149	100	1.56	42.9	5.19	36.6
Catalan	547,261	100	1.36	31	4.9	29.9
Chinese	186,619	100	2.1	85.4	4.6	24.3
English	742,304	100	1.25	20.7	6.18	72.5
Estonian	528,411	100	1.78	55.6	6.51	91.4
Finnish	396,999	100	1.86	59.6	6.62	98.1
French	635,081	100	1.39	32.5	5.44	43.5
German	3,810,131	100	1.54	34.4	6.08	67.6
Greek	88,934	100	1.59	38.4	4.52	22.9
Hindi	351,704	100	1.38	26.3	4.57	23.8
Indonesian	169,728	99.99	1.37	30.5	6.07	67.2
Italian	879,629	99.993	1.39	30.3	6.39	83.7
Japanese	222,442	100	2.19	91.9	3.83	14.2
Korean	446,996	100	2.26	74.4	5.15	35.5
Portuguese	1,464,445	100	1.38	32.1	6.54	93.3
Russian	1,830,033	99.998	1.59	46.4	5.34	40.6
Spanish	1,023,080	99.997	1.31	26.4	5.44	43.3
Turkish	681,936	100	1.74	56	7.25	152
Urdu	138,077	100	1.38	26.8	5.05	33.2
Vietnamese	59,957	100	1.46	37.8	6.22	74.5

## Appendix C. Eligible Dependency Spans by Language and Distance

**Table A3:** Eligible dependency-span counts at distances 4–10.

Language	d = 4	5	6	7	8	9	10	Total
Arabic	5738	3234	2249	1366	1092	730	526	14,935
Basque	3814	2085	1237	695	424	234	135	8624
Bulgarian	4906	2514	1393	857	507	310	215	10,702
Catalan	19,701	11,028	6704	4423	2983	2082	1515	48,436
Chinese	7429	4704	3024	1888	1247	879	500	19,671
English	35,594	17,023	9451	5748	3568	2201	1428	75,013
Estonian	21,638	12,318	6899	3895	2225	1228	724	48,927
Finnish	14,773	7292	3727	2016	1056	609	337	29,810
French	20,022	10,736	6443	3830	2664	1736	1154	46,585
German	149,523	100,024	77,572	60,481	46,848	35,390	26,468	496,306
Greek	3134	1628	1059	595	379	240	162	7197
Hindi	10,371	7718	6412	5545	4725	4124	3419	42,314
Indonesian	4816	2656	1583	939	597	364	241	11,196
Italian	28,296	14,154	8713	5416	3628	2434	1731	64,372
Japanese	8269	4232	2277	1509	1004	748	536	18,575
Korean	14,135	9244	6464	4849	3708	2757	2051	43,208
Portuguese	44,529	22,544	13,299	8307	5194	3575	2400	99,848
Russian	47,819	23,689	12,301	6581	3629	2097	1229	97,345
Spanish	33,373	19,187	11,544	7101	4994	3357	2180	81,736
Turkish	21,255	13,611	8874	6271	4076	2851	2040	58,978
Urdu	4511	3324	2791	2248	2005	1819	1506	18,204
Vietnamese	1806	967	597	337	190	148	95	4140
All languages	505,452	293,912	194,613	134,897	96,743	69,913	50,592	1,346,122
